# HDAC6 Regulates the Chaperone-Mediated Autophagy to Prevent Oxidative Damage in Injured Neurons after Experimental Spinal Cord Injury

**DOI:** 10.1155/2016/7263736

**Published:** 2015-11-15

**Authors:** Min Su, Huaqing Guan, Fan Zhang, Yarong Gao, Xiaomei Teng, Weixin Yang

**Affiliations:** ^1^Department of Rehabilitation, First Affiliated Hospital of Soochow University, Suzhou 215006, China; ^2^Institute of Neuroscience, Soochow University, Suzhou 215123, China; ^3^Department of Orthopaedics, First Affiliated Hospital of Soochow University, Suzhou 215006, China; ^4^Institute for Cardiovascular Science, Soochow University, Suzhou 215006, China

## Abstract

Hypoxia-ischemia- (HI-) induced oxidative stress plays a role in secondary pathocellular processes of acute spinal cord injury (SCI) due to HI from many kinds of mechanical trauma. Increasing evidence suggests that the histone deacetylase-6 (HDAC6) plays an important role in cell homeostasis in both physiological and abnormal, stressful, pathological conditions. This paper found that inhibition of HDAC6 accelerated reactive oxygen species (ROS) generation and cell apoptosis in response to the HI. Deficiency of HDAC6 hindered the chaperone-mediated autophagy (CMA) activity to resistance of HI-induced oxidative stress. Furthermore, this study provided the experimental evidence for the potential role of HDAC6 in the regulation of CMA by affecting HSP90 acetylation. Therefore, HDAC6 plays an important role in the function of CMA pathway under the HI stress induced by SCI and it may be a potential therapeutic target in acute SCI model.

## 1. Introduction

Spinal cord injury (SCI) is a kind of serious and debilitating disease. The main clinical manifestations of SCI are neurological dysfunction at and/or below the level of the injury [1]. The disability and lethal rate of this disease are extremely high and at present there is no effective treatment to it [1]. Although the underlying pathocellular processes of SCI remain uncertain, secondary damage following primary SCI extends pathology beyond the site of initial trauma, characterized by neurons inflammation, demyelination, and axonal degeneration, and various degrees of oligodendrocyte and neuronal cell death [2]. Consequently, defining the mechanism of secondary damage will be important to understand neurodegenerative disorders and find the best therapeutic procedures.

Hypoxia-ischemia (HI) of the cord, resulting from various mechanical trauma, has been reported to induce the formation of active oxygen and free radicals (reactive oxygen species, ROS) which can bring irreversible secondary lesion [3, 4]. In another word, SCI is considered to be related to a vulnerability of spinal somatic and motor neurons to HI as well as the involvement of ROS [5]. However, the mechanisms underlying this vulnerability are not fully understood.

Several reports have described that autophagy occurred in SCI [6, 7]. Three different types of autophagy have been described in mammalian cells; chaperone-mediated autophagy (CMA) is one type of autophagy that was involved in resisting the ROS-induced motoneuronal death during spinal cord development [8, 9].

In addition, the recent evidence directly supports that the knockdown of histone deacetylase-6 (HDAC6) triggered a significant generation of ROS and disruption of mitochondrial membrane potential (MMP) [10]. Several investigations have demonstrated that targeting HDAC6 activity can protect neurons and glia and improve outcomes in CNS injury and disease models [11–13]. However, the role of HDAC6 in acute SCI remains unclear.

## 2. Materials and Methods

### 2.1. Animals and Surgical Procedures

A total of 30 adult female C57BL/6J mice (10–12 weeks old; Laboratory Animals Center of the Medical College of Soochow University, Suzhou, China) were used in this study. Each experimental group includes at least 5 mice. Every cage housed three or four mice and the temperature was kept at 24°C. All of the animals easily get enough water and food before and after surgery.

The mice were anesthetized with 1.25% halothane in an oxygen/nitrous oxide (30/70%) gas mixture. During surgery the rectal temperature was monitored and maintained at 37.0 ± 0.5°C by a heating pad. A sterile manner was used to preserve the skin above the thoracic vertebrae and 15 mm midline skin incision was made. Then the laminae of T7–9 were exposed, and the laminectomy was performed at T8 till the dura mater emerged. With a sharp scalpel, the spinal cord was hemitransected on the right side only [14]. Finally, the muscles and skin were closed in layers. The mice with compromised bladder function (a rare complication) received manual bladder expression twice a day until establishing reflex bladder emptying. The same surgical procedures were performed to the sham operated animals, but without the hemisection to the spinal cord. All surgical and animal handling procedures were performed following the guidelines of the National Institutes of Health for the Care of Animals, approved by the Experimental Animal Center, Soochow University, Suzhou, China.

### 2.2. Cell Culture and Treatments

The rat pheochromocytoma (PC12) cell line was provided by Shanghai Institute of Cell Biology, Chinese Academy of Sciences. Cells were maintained in complete Dulbecco's modified Eagle's medium (DMEM), supplemented with 10% fetal bovine serum (FBS) and 1% penicillin/streptomycin. In order to simulate hypoxia-ischemia (HI) condition, the cultures were transferred to a serum-free medium (only DMEM) preequilibrated with 95% N_2_ and 5% CO_2_. Then the cells were incubated in the medium and placed in the incubator equipped with 3% O_2_.

### 2.3. Transient Transfection with HDAC6 siRNA

Three interference sequences were designed and tested for HDAC6 silencing by Shanghai Zimmer. Silencing efficiency of two interference sequences was more than 70%. The two interference sequences were chosen for further experimentation based on their ability to block HDAC6 expression. The PC12 cells were transiently transfected with HDAC6 siRNA or control sequence using Lipofectamine 2000 (Invitrogen) in accordance with the manufacturer's instructions. At 24 h after transfection, cells were subjected to Western blotting analysis. And GFP immunofluorescence was also assessed with an inverted fluorescence microscope. 48 h later, the cells with the greatest reduction of HDAC6 expression were used for further studies. Specific methods were showed in the previous publication [15].

### 2.4. Histological Assessments

100 mg/kg sodium pentobarbital was injected to the mice by abdominal cavity at 24 h after the operation. The normal saline was overdosed to the mice by transcardial perfusion, followed by 4% paraformaldehyde in 0.1 M PBS, pH 7.4. Before the immunohistochemical staining, the spinal cord segments containing the injured center were collected, postfixed in the same fixative overnight at 4°C, and embedded in paraffin [14]. Serial 1 cm segment of spinal cord including the injury sites was dissected and sectioned longitudinally in the horizontal plane or transversely at 5 *μ*m thickness on the slides. The sections were used for immunohistochemical and TUNEL staining. TUNEL staining was used to observe neuronal apoptosis, and SP staining was used to observe the HDAC6 expression in the neuron. Then the positive expression units were semiquantitatively analyzed using the Image-pro plus software which used surface density and optical density [16].

### 2.5. Western Blotting Analysis

Briefly, a 10 mm spinal cord segment containing the injury center was removed for protein extraction 24 h following SCI. After each indicated treatment, equal amounts of protein from the cell or tissue extracts were mixed with sodium dodecyl sulphate polyacrylamide gel electrophoresis (SDS-PAGE) sample buffer and boiled for 8 min. Equal amounts of proteins (15 *μ*g) were loaded and separated by SDS-PAGE in Tris-glycine running buffer. After that, proteins were transferred onto a polyvinylidene difluoride membrane (Millipore, Bedford, MA, USA) and blocked with 5% nonfat milk in Tris-buffered saline for 30 min. The membranes were then incubated with anti-HDAC6 (1 : 100; Biovision), anti-LAMP-2a (1 : 1000; Abcam), anti-HSP90 (1 : 1000; Abcam), anti-HSC70 (1 : 1000; Abcam), and anti-HIF-1-alpha antibody (1 : 500; Abcam) at 4°C overnight. After washing in Tris-buffered saline containing 0.1% Tween 20 (TBST) for 3 times, the membranes were incubated with horseradish peroxidase-conjugated secondary antibodies (Vector Laboratories, Burlingame, CA) in TBST containing 3% nonfat dry milk for 2 h. Immunoreactivity was detected with enhanced chemiluminescence autoradiography (ECL kit, Amersham, Arlington Heights, IL). The densitometry of the bands was quantitatively analyzed with Sigma Scan Pro 5 software (NIH, Bethesda, MD, USA). Independent experiments were carried out in triplicate. *β*-actin was used as protein loading control. Repeat 3 times.

### 2.6. Immunoprecipitations

At the end of treatment, the culture media were aspirated and the cells were washed once with ice-cold PBS. The cells were then lysed with lysis buffer containing 20 mM Tris (pH 7.5), 150 mM NaCl, 1% Triton X-100, and sodium pyrophosphate, *β*-glycerophosphate, EDTA, Na_3_VO_4_, leupeptin, and other protease inhibitors (Sigma-Aldrich). Protein samples per 100 *μ*g were added with 4 *μ*L anti-HSP90 antibody (Abcam, Cambridge, UK) and shaked at 4°C overnight. 40 *μ*L Protein A Agarose (Sigma Chemical Company) was added and incubated for 3 h at 4°C and then centrifuged at 1000 g for 5 min. After that, the supernatant was discarded and the pellet was washed 3 times with PBS. Thereafter, the precipitates were resuspended with 40 *μ*L 1x sample buffer and heated at 96°C for 5 min. The samples were centrifuged for 5 s and the supernatants were collected for Western blotting.

### 2.7. Intracellular ROS Determination

Reactive Oxygen Species Assay Kit was obtained from Sigma Chemical Company (D6883, USA). Intracellular ROS was measured using the nonfluorescent probe 2′,7′-dichlorofluorescein diacetate (DCFH-DA). PC12/siRNA PC12 cells were plated at a density of 1 × 10^5^/well in 6-well plates. One day after plating, the cells were treated with serum-free and hypoxia (3% O_2_) for 24 h. For specific method, refer to the previous publication [17]. After DCFH-DA treatment, the cells were washed with cold phosphate-buffered saline (PBS), collected, and subjected immediately to flow cytometry (Becton Dickinson FACSCalibur) analysis of DCF fluorescence at excitation wavelength of 488 nm and emission wavelength of 610 nm. The fluorescence was expressed as a percentage of total area. This process was repeated 3 times.

### 2.8. ELISA

ELISA kits for rat RNase A were obtained from Antibodies Company (ABIN431684, Aachen, Germany). Cells were collected after treatment, then immunoprecipitation of RNase A from cells as the manufacturer's instructions. Briefly, the supernatants were mixed with anti-RNase A antibody (1 : 5000; Rockland, PA, USA) and incubated with Protein A Agarose beads. After appropriate washing, aspirate last wash and proceed as all ELISA methods. The fluorescent properties of each sample and appropriate standards were measured using a Microplate Reader, read absorbance at 450 nm. The data were linearized by plotting the log of the RNase-A concentrations versus the log of the OD and the best fit line was determined by curve expert 13.0. This process was repeated 3 times.

### 2.9. Immunofluorescence Microscopy

The cells were collected and washed by PBS for 3 × 5 min, followed by being fixed for 20 min in PBS containing 4% paraformaldehyde (pH 7.4). After that, the cells were washed in PBS for 4 × 5 min and blocked in PBS containing 1% normal bovine serum albumin and 0.1% Triton-X-100 for 10 min at room temperature. Then we exposed the cells to anti-LAMP-2a (1 : 200; Abcam), anti-LAMP-1 (1 : 200; Abcam), or anti-HDAC6 (1 : 250; Abcam) antibody at 4°C overnight. Cultures were subsequently washed and incubated with mixture secondary antibody at 37°C for 1 h and then rinsed several times and incubated again with 10 mg/mL 4-6-diamidino-2-phenylindole (DAPI; Serva, Heidelberg, Germany) for 10 min at room temperature. At last, cultures were mounted on glass slides with Vectashield mounting medium (Vector Lab) and analyzed with a confocal microscope (LEICA TCS SP5II, Germany). Images were digitally analyzed by Leica microsystem software to quantify the fluorescence intensity of cells. From each group, 5 pieces of coverslips including at least 60 cells were analyzed.

### 2.10. Cells Apoptosis Analysis

Apoptosis was measured by flow cytometry to detect annexin V staining and propidium iodide uptake (Invitrogen Detection Technology, Eugene, OR) as described previously [15]. Three independent experiments were performed to determine the standard deviation.

### 2.11. Transmission Electron Microscopy

After each treatment, cells were harvested and viewed under a transmission electron microscope (JEM-1011, Japan). Specific methods were showed in the previous publication [15].

### 2.12. Statistical Analysis

All experiments were performed for at least three sets of independent experiments. The data are presented as mean ± SEM. Two group comparisons were performed using Student's *t*-test. Multiple group comparisons were performed using one-way analysis of variance and Fisher's least significant difference. A *P* value of less than 0.05 was set as statistically significant.

## 3. Results

### 3.1. The Spinal Cord Injury Activates the HDAC6 and CMA

**Figure 1:**
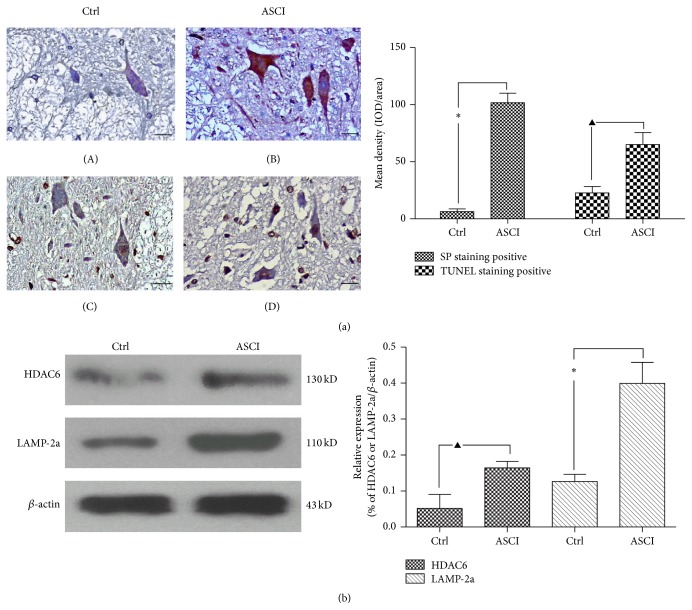
The expression of HDAC6 and LAMP-2a protein increased in the ASCI. 30 adult female C57BL/6J mice were divided into two groups: the T8 spinal cord hemitransected group (ASCI) and the sham operated group (Ctrl). (a) The expression of HDAC6 was analyzed by SP standing at 24 h after the operation ((A)-(B)). Brown represents HDAC6 positive expression in the cytoplasm, and its negative cell cytoplasm is light blue, while gray matter supports network negative staining; ((C)-(D)) TUNEL staining was used to observe neuronal apoptosis. (C) Field of vision is a small amount of TUNEL staining positive neurons and glial cells, which shows that only a small amount of neurons is undergoing apoptosis. Spinal cord tissue structure is basic intact, and there are few cavity formations. (D) Field of vision is more TUNEL staining positive neurons and glial cells (tan for its nucleus is positive), which shows that a large number of nerve cells are undergoing apoptosis. The structure of the spinal cord tissue is disorder, and there are more cavity formations. Scale bar: 20 *μ*m. Semiquantitative analysis are consistent with the figure, and HDAC6 expression in ASCI was dramatically increased compared with the control group. At least 60 cells were included for analysis from five images per group. Values represent mean ± SEM, ^*∗*^ *P* < 0.01, ^▲^ *P* < 0.05 versus corresponding control group. (b) By Western blot, in the ASCI group, the protein expression of HDAC6 and LAMP-2a was significantly higher than the control group. Values represent mean ± SEM, ^*∗*^ *P* < 0.01, ^▲^ *P* < 0.05 versus corresponding control group. Each experiment repeated at least 3 times.

In order to confirm the role of HDAC6 and CMA in acute SCI, a spinal cord hemitransected model in mice was set up. As indicated in Figure 1(a), after acute SCI for 24 h, HDAC6 protein expression in the damaged spinal cord tissue was dramatically increased compared with the sham operated group (*P* < 0.01). SP staining showed that the presence of brown granules was found in some neurons' cytoplasm after injury versus sham whereas the negative neurons become pyknotic (Figure 1(a)). TUNEL staining data further demonstrated the greater number of apoptosis neurons in the damaged spinal cord tissue compared to the sham operated group at 24 h after being hemitransected (*P* < 0.05). From these results, we confirmed that acute SCI caused the increased HDAC6 expression, which is associated with neuronal apoptosis in the damaged spinal cord.

In Western blot experiments, we further found that not only the protein expression of HDAC6, but also the protein expression of LAMP-2a was increased in the SCI mice compared with sham operated mice (*P* < 0.05) (Figure 1(b)). The previous studies had shown that the LAMP-2a is the speed-limiting protein of the CMA metabolic pathway [18]. Therefore, we speculated that HDAC6 affects the neuronal survival by regulating the CMA activity. In order to confirm this hypothesis, a series of* in vitro* studies were conducted.

### 3.2. Hypoxia-Ischemia Induced HDAC6 Expression and Oxidative Stress* In Vitro*

It is well known that various mechanical trauma would induce the hypoxia-ischemia (HI) which is the primary cause of secondary damage of SCI and can bring irreversible neurodegenerative changes [2]. To replicate the HI pathological state, the PC12 cells were treated with serum-free and hypoxia (3% O_2_) for 24 h.

**Figure 2:**
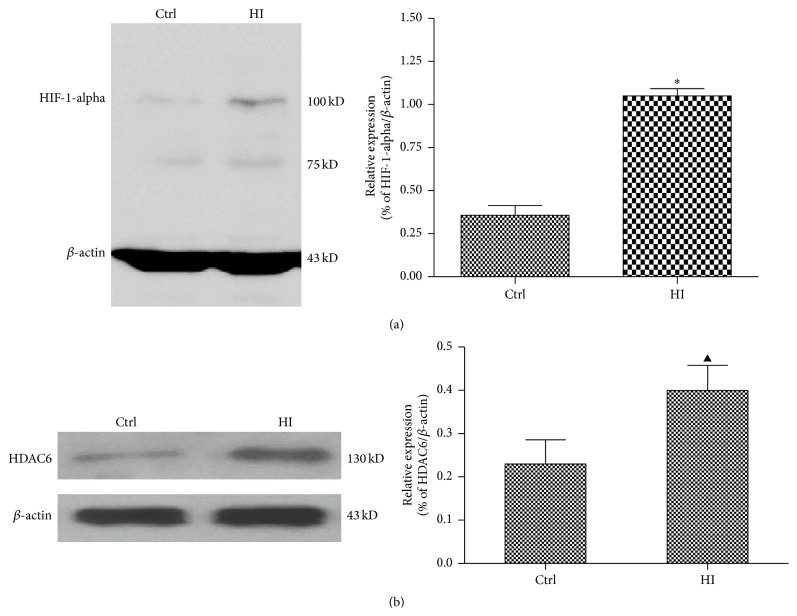
Hypoxia-ischemia induced HDAC6 expression and oxidative stress* in vitro*. The PC12 cells were treated with HI for 24 h. Ctrl express medium-treated group. (a) The expression of HIF-1-alpha was analyzed by Western blot. Results of the densitometric quantification are represented as mean ± SEM (*n* = 3). ^*∗*^ *P* < 0.01 versus control group. (b) The expression of HDAC6 was analyzed by Western blot. Results of the densitometric quantification are represented as mean ± SEM (*n* = 3). ^▲^ *P* < 0.05 versus control group.

From Figure 2(a), HI cause the protein expression of hypoxia-inducible factor 1 alpha (HIF-1-alpha) dramatically increase compared with the medium-treated group (*P* < 0.01). While the reactive oxygen species (ROS) is able to promote rapid activation and stabilization of the transcription factor HIF-1-alpha, which regulates expression of genes involved in inflammation, metabolism, and cell survival [19]. Therefore these evidences suggested to us that HI triggered the generation of ROS and PC12 cells were actually experiencing hypoxia.

By exposed PC12 cell in HI condition for 24 h, the protein expression of HDAC6 was also significantly increased compared with medium-treated group (*P* < 0.05) (Figure 2(b)). It has been proved that the accumulation of ROS is an oxidative stress to which cells respond by activating various defense mechanisms; thereby we speculated that HDAC6 is involved in the process of the cells resistance to oxidative stress. This needs further study to approve the hypothesis.

### 3.3. Deficiency of HDAC6 Accelerates the Oxidative Stress in Response to Hypoxia-Ischemia

To investigate whether HDAC6 is essential for the cells against ROS-induced cytotoxicity,* in vitro* flow cytometry studies were applied to detect the intracellular level of ROS.

**Figure 3:**
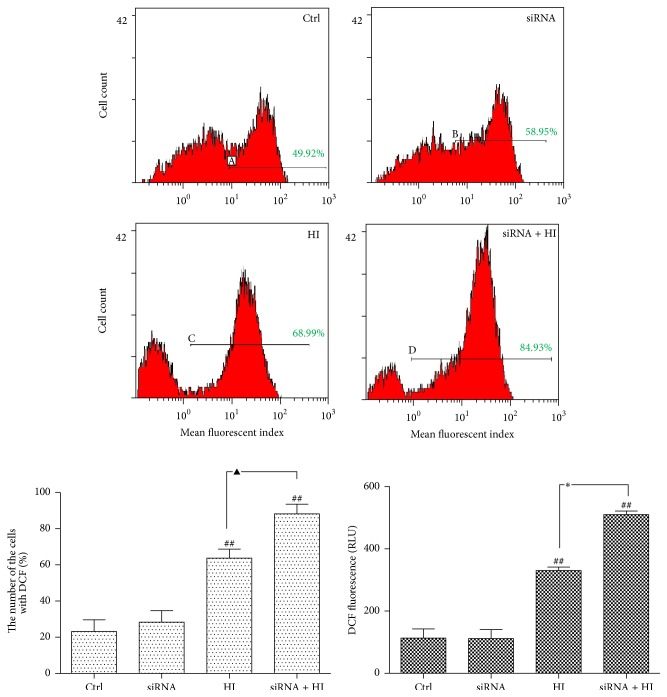
Deficiency of HDAC6 accelerates intracellular ROS generation in response to HI. The PC12 and PC12/siHDAC6 cells were treated with HI for 24 h. Ctrl express vehicle-treated group. Changes in the intracellular DCF fluorescence value and the percentage of cell with fluorescence can be detected by flow cytometry. The *Y*-axis indicates the number of cells with fluorescence; the *X*-axis expresses fluorescence intensity. Red indicates the level of intracellular ROS. Green indicates the figure of %Gated. Result of one representative experiment was shown. The densitometric quantification is represented as mean ± SEM (*n* = 3), ^##^ *P* < 0.01 versus control group; ^*∗*^ *P* < 0.01, ^▲^ *P* < 0.05 indicate the significance among the groups indicated in the bar chart.

As shown in Figure 3, HI induces a net increase of intracellular ROS compared with the vehicle-treated cells (*P* < 0.01), which is reflected by the DCF fluorescence value and the percentage of cell with fluorescence. After the siRNA inhibition of HDAC6, the generation of ROS was further accentuated to the HI-induced stress. These findings suggest that hypoxic stress induces accumulation of ROS in neuronal cells whereas HDAC6 deficiency induced the occurrence of the secondary oxidative stress which is considered to contribute to neural tissue damage. However, disruption of HDAC6 stimulates ROS production which may be related to the decreased cellular antioxidant activity.

### 3.4. Inhibition of HDAC6 Aggravates Cell Apoptosis to HI-Induced Oxidative Stress

Oxidative stress is one of the primary metabolic disorders jeopardizing cell survival [20]. To investigate whether HDAC6 is essential for the cells against ROS-induced cytotoxicity, the apoptosis of PC12 cells was analyzed by flow cytometry.

**Figure 4:**
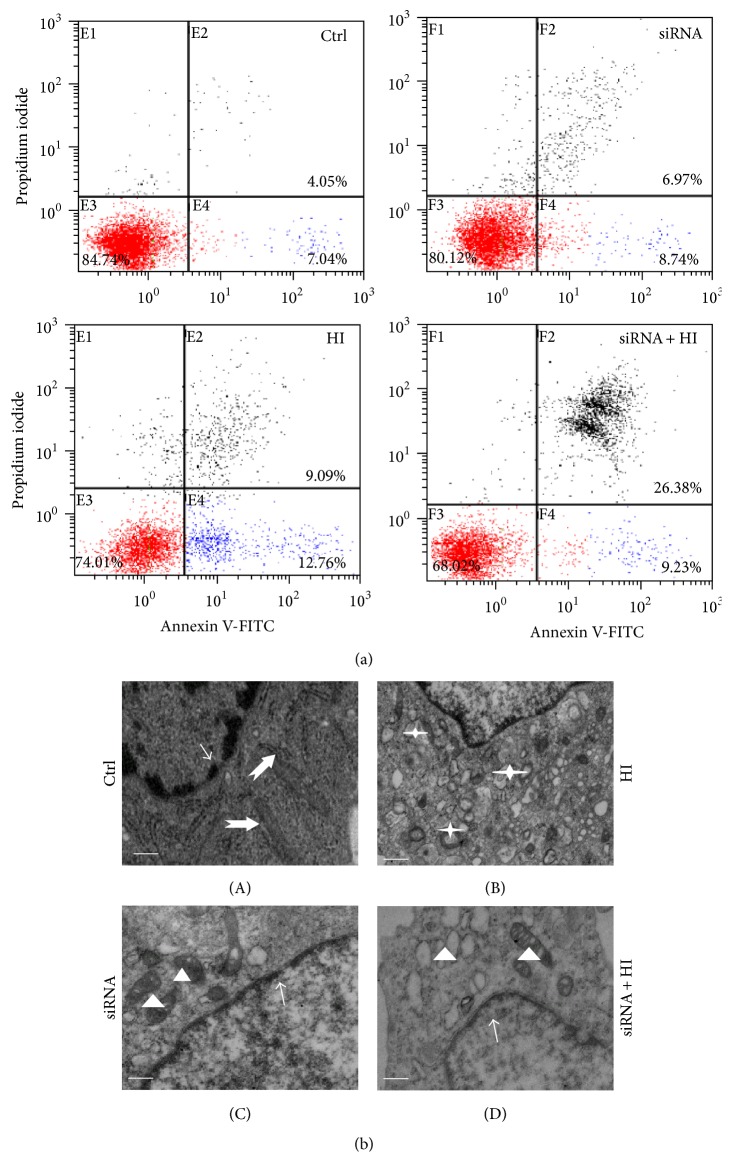
Loss of HDAC6 leads to the hypersensitivity to HI-induced stress. The PC12 and PC12/siHDAC6 cells were treated with HI for 24 h. Ctrl express vehicle-treated group. (a) Apoptotic cells were calculated by flow cytometry with simultaneous staining of annexin V-FITC and PI. (b) The ultrastructural features of PC12 cells in different groups. In control cells (A) and HDAC6-deficient cells (C), the nucleoplasm (thin arrows) remained condensed and all kinds of organelles scattered within the cytoplasm (thick arrows and triangles). HI treatment induced autophagy (B); the lysosome amplification and autophagy bubbles appear in the cytoplasm (asterisks). (D) Knockdown of HDAC6 elicited apoptotic features in cells exposure to HI. Cell swelling, cytoplasmic vacuolization (triangles), and nuclear chromatin loss and margination (thin arrows). Scale bar: 1 *μ*m.

An increase in the number of apoptotic cells, especially early apoptosis, occurred after PC12 cells were exposure to HI for 24 h (Figure 4(a)). Knockdown of HDAC6 resulted in a slow but evidently increased rate of apoptosis reflected to HI (Figure 4(a)). Furthermore, the ultrastructural features of PC12 cells were examined with transmission electron microscopy (EM). Compared with vehicle-treated control cells (Figure 4(b)-(A)), HI-treated cells exhibited the initial characteristics of autophagy, such as lysosome amplification and autophagy bubbles in the cytoplasm (Figure 4(b)-(C)). EM studies confirmed that knockdown of HDAC6 further elicited cell apoptosis in response to HI. As shown in Figure 4(b)-(D), nuclear chromatin margination, cytoplasmic vacuolization, and the apoptotic bodies appeared. These results showed that HI triggered early cell apoptosis, and deficiency of HDAC6 increased the sensitivity of cells to HI-induced stress, which can exacerbate the cellular damage and apoptosis. Meanwhile the form of apoptosis further causes the production of ROS, eventually leading to irreversible cell death.

### 3.5. HDAC6 Regulates the Activity of CMA Degradation Pathway in Response to Hypoxia-Ischemia

To clarify the mechanism of deficiency of HDAC6 leading to cell apoptosis and ROS increase, the further study was made. The recent evidence directly supports the fact that the CMA was involved in resisting the ROS-induced motoneuronal death during spinal cord development [21, 22]. Thereby, we observed the effect of HDAC6 on the CMA activity.

**Figure 5:**
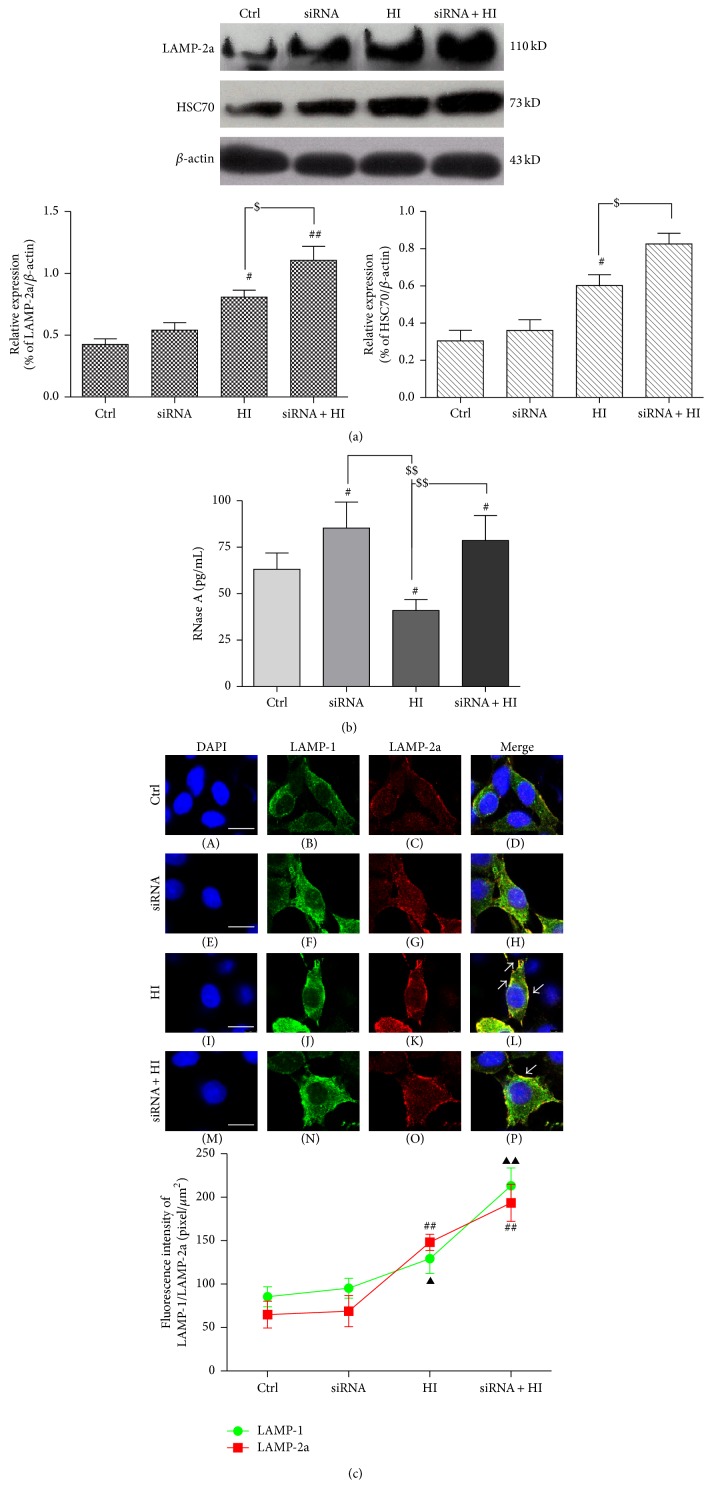
Deficiency of HDAC6 disrupts the CMA activity in response to HI. The PC12 and PC12/siHDAC6 cells were treated with HI for 24 h. Ctrl express vehicle-treated group. (a) The expression of LAMP-2a and HSC70 was analyzed by Western blot. The level of LAMP-2a and HSC70 was further observed in HDAC6 knockdown cells by siRNA. Results of the densitometric quantification are represented as mean ± SEM (*n* = 3). ^#^ *P* < 0.05, ^##^ *P* < 0.01 versus control group; ^$^ *P* < 0.01 indicates the significance among the groups indicated in the bar chart. (b) Immunoprecipitated RNase A (an identified CMA substrate) from the treated cells and then assay RNase A activity by ELISA. Group data represent mean ± SEM (*n* = 3); ^#^ *P* < 0.05 versus corresponding control group; ^$$^ *P* < 0.01 indicates the significance among the groups indicated in the bar chart. (c) Double immunostained with antibody to LAMP-1 (green) and LAMP-2a (red) as indicated. Superimposed confocal images (merge) demonstrate the colocalization of LAMP-2a with LAMP-1 (a lysosome marker). Arrows indicate LAMP-2a mainly clustered in the perinuclear lysosomal membrane. Scale bar: 15 *μ*m. The fluorescence intensity of LAMP-1 and LAMP-2a from experiments results shown. At least 60 cells were included for analysis from five images per group. Group data represent mean ± SEM (*n* = 3); ^▲^ *P* < 0.05, ^▲▲^ *P* < 0.01, and ^##^ *P* < 0.01 versus corresponding control group.

The HSC70 and LAMP-2a, two relatively specific markers of CMA, were examined and semiquantified by Western blotting analysis. The expression level of both proteins was significantly increased in the HI-treated cells compared with the vehicle-treated cells (Figure 5(a)). However, it was interesting that inhibition of HDAC6 by siRNA further increased the expression of LAMP-2a and HSC70 in PC12 cells exposed to HI for 24 h (Figure 5(a)). In order to confirm whether the CMA activity was consistent with the changes of LAMP-2a and HSC70, the amount of RNase A, a well-characterized protein substrate of CMA proteolytic pathway, was examined [23]. Of note, HI induced the increase in substrate degradation whereas an opposite change was observed if HDAC6 was inhibited (Figure 5(b)). These results suggest that HI may activate CMA degradation pathway which may be responsible, at least in part, for the accumulation of damaged abnormal and unnecessary proteins induced by oxidative stress in the cytoplasm. Inhibition of HDAC6 caused the reduction of CMA activity, and the increase of LAMP-2a and HSC70 may only act as a compensatory response to the ROS-induced stress.

Furthermore, we confirmed the change of LAMP-2a and LAMP-1, a marker of lysosome [24], by the immunofluorescence staining. As shown in Figure 5(c), HI treatment significantly increased the expression of LAMP-2a and also enhanced the colocalization of LAMP-2a with LAMP-1 around the nucleus. Simultaneously, the increase of LAMP-2a tends to be more notable than that of LAMP-1. Of interest, after deficiency of HDAC6, the level of LAMP-1 was shown to be further enhanced and significantly exceeded the LAMP-2a, while the colocalization of LAMP-2a with LAMP-1 did not add (Figure 5(c)). Therefore, it could be speculated that HI indeed activate CMA degradation pathway, and inhibition of HDAC6 induced LAMP-2a which is compensatory increased partially by the expansion of lysosomal membrane, instead of further improving the CMA degradation activity to resist HI-induced oxidative stress toxicity.

### 3.6. HDAC6 Affects the Activity of CMA by Regulating HSP90 Acetylation

Why does the inhibition of HDAC6 disrupt the activity of CMA? Previous evidence indicates that HSP90, as a constitutively and ubiquitously expressed ATP-dependent molecular chaperone, was also associated with lysosomes and, thus, may play critical roles in the functional dynamics of the CMA [25, 26]. As HDAC6 deacetylates lysine residues of HSP90 [27], the further study was continued to investigate whether HDAC6 may affect the activity of CMA by regulating the acetylation of HSP90.

**Figure 6:**
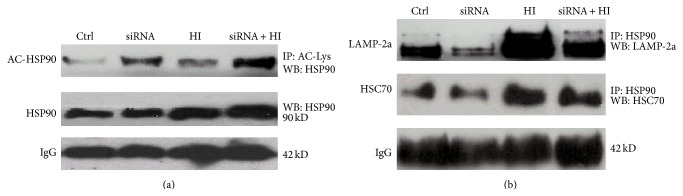
HDAC6 affects the activity of CMA by regulating the level of acetylated HSP90. (a) The cell lysates isolated from PC12 and PC12/siHDAC6 cells exposed to HI for 24 h. Ctrl express vehicle-treated group. The protein complexes were immunoprecipitated with anti-acetylation antibody and blotted with anti-HSP90 antibody for determination of acetylated HSP90 (Ac-Hsp90) level. The level of IgG was used as a loading control. (b) The immunocomplexes were precipitated from PC12 and PC12/siHDAC6 cells with anti-HSP90 antibody and blotted with anti-HSC70 or anti-LAMP-2a antibodies for determination of the interaction of HSP90 with HSC70 or LAMP-2a. The level of IgG was used as a loading control.

To this end, the level of acetylated HSP90 and the interaction of HSP90 with HSC70 and LAMP-2a in response to HI were analyzed in combination with HDAC6 siRNA and immunoprecipitation techniques. As shown in Figure 6(a), after HI treatment for 24 h in PC12 cells, the total level of HSP90 was markedly increased, while HSP90 acetylation level did not show any significant change. However, the level of acetylated HSP90 was significantly increased by the inhibition of HDAC6. Moreover, in HDAC6 deficient cells, the interaction of HSP90 with LAMP-2a and HSC70 was markedly decreased in response to HI (Figure 6(b)). These results strongly suggest that HDAC6 may affect the activity of CMA by acting as a HSP90 deacetylase. As a key molecule chaperone to resist the oxidative stress [28], the expression of compensatory HSP90 increases after loss of HDAC6, and increased HSP90 protein synthesis may determine its greater resistance to stressor that elicits the formation of ROS-induced cellular damage.

## 4. Discussion

The present study firstly revealed SCI induced HDAC6 expression increase* in vivo*; simultaneously inhibition of HDAC6 accelerates ROS generation and neurons apoptosis in response to the hypoxia-ischemia (HI)* in vitro*. Second, a positive correlation between HDAC6 and CMA was represented* in vivo* and* in vitro*, and inhibition of HDAC6 hinders the CMA activity. Third, our results provided the experimental evidence for the potential role of HDAC6 in the regulation of CMA by affecting HSP90 acetylation.

Effective management of the secondary damage following primary SCI is imperative for maximizing anatomical and functional recovery [29]. Consequently, defining the mechanism of secondary damage will be important to the understanding of neurodegenerative disorders and to find the best therapeutic procedures. In recent years, several laboratories have obtained experimental evidence indicating that oxidative stress elicited by HI from all kinds of mechanical trauma is a major player in the pathogenesis of secondary damage after acute SCI [30–32]. The involvement of ROS in neuronal subsequent death has been determined, which can induce about 50% of the neuronal programmed cell death [30]. Our study shows that overproduction of ROS was correlated in a significant manner with the apoptotic index (Figures 3 and 4). High levels of ROS can oxidize cell constituents, such as lipids, proteins, and DNA, thus posing a threat to the cell integrity and viability [33]. Various defense mechanisms have been mobilized to protect cells against oxidative stress [34].

Our data had shown that both SCI and HI caused the expression increase of HDAC6 (Figures 1 and 2). As a member of the histone deacetylase family, HDAC6 is mainly localized in the cytoplasm and has two catalytic sites and an ubiquitin-binding domain at the C terminus [35]. Due to the special structure and positioning of HDAC6, it has been implicated to be involved in many cellular processes, including degradation of misfolded proteins, cell migration, and cell-cell interaction [36]. The reduction of HDAC6 activity in cultured cells may compromise cell viability when the cells are exposed to different stressors. Recently, malfunctioning of HDAC6 has been linked to a growing number of human disorders [37, 38]. In this study, we also provided the evidence that inhibition of HDAC6 accelerates ROS generation and neurons apoptosis in response to the hypoxia-ischemia (HI)* in vitro* (Figures 3 and 4).

To clarify the mechanism of HDAC6 in secondary damage following primary SCI, we observed the changes in the acetylation level of Hsp90 which is not only the exclusive substrate of HDAC6, but also a critical molecular chaperone to CMA degradation pathway. The dynamic equilibrium of protein acetylation and deacetylation plays a pivotal role in the normal physiological process of cells. Acetylation of HSP90 at lysine294 has been shown to modulate its activity by regulating client protein and cochaperone binding [39]. In addition, our study showed that the expression of compensatory HSP90 increases after loss of HDAC6 in HI condition. As a key molecule chaperone to resist the oxidative stress [28], increased HSP90 protein synthesis may determine its greater resistance to stressor that elicits the formation of ROS-induced cellular damage.

Our findings signaled that SCI increases the components of the CMA pathway and a positive correlation between HDAC6 activity and indicators of CMA pathway* in vivo* and* in vitro* (Figures 1 and 5). In mammalian cells, the pathway of CMA is constitutively and maximally activated under stressful conditions, especially oxidative stress [22]. As a cell repair mechanism, CMA was really activated during HI in our study (Figure 5). The pathway of CMA selectively degrades cytosolic proteins containing the KFERQ (five peptide sequences can be recognized by a chaperone complex containing HSC70) signal motif through direct translocation into the lysosome [40]. Oxidized substrate proteins are translocated into lysosomes more efficiently by this pathway than marcoautophagy [33]. In addition, under mild oxidative stress condition, the lysosomes bring into play a higher tendency to bind and internalize substrates transferred by CMA [7, 33]. Collectively, this shows that CMA provides the front line of defense against oxidative stress [33]. Therefore, we believe that CMA favors degradation of abnormal and unnecessary proteins against that of proteins essential for cell survival. This may also explain why CMA degradation pathway can be activated under SCI and hypoxia-ischemia condition.

The successful implementation of the CMA depends on substrates, which could be recognized by a chaperone complex containing HSC70 and delivered into lysosomes via the interactions with LAMP-2a. The cycle of HSC70-client protein complex involves successive association and dissociation with chaperone HSP90 to form various multimeric protein complexes [41]. This is dictated by the ATP-binding state of HSP90 [42]. A conformational change in HSP90 leads to the release and realignment of cochaperones HSC70 to form a mature chaperone-subtract complex [41–43]. The change of HSP90 activity is closely related to its acetylation level. Our study demonstrates for the first time that the depletion of HDAC6 levels led to the increase of HSP90 acetylation and decrease of the association between HSC70 and HSP90 in cells exposure to HI (Figure 6).

Moreover, the chaperone-substrate complex must specifically bind to LAMP-2a which located on the membrane of the lysosomal, a receptor for CMA, and the binding of substrates to LAMP-2a is the limiting step for CMA [18]. The substrate only binds with LAMP-2a monomers and promotes the organization of LAMP-2a into a high multimeric complex of approximately 700 kDa, a critical step for substrate translocation [18, 23]. When CMA is maximally activated, the level of LAMP-2a located on the lysosomal membrane may increase [23, 24]. The LAMP-2a undergoes continuous cycles of assembly/disassembly, while HSP90 is the key molecule chaperone to drive the cycle. HSP90 binds to the lysosomal membrane to stabilize LAMP-2a and induce it to change from the monomeric to the multimeric state in order to efficaciously transfer substrate proteins [23, 26]. Our results showed that knockdown of HDAC6 induced irreversible hyperacetylation of HSP90 and attenuated the interaction of HSP90 with LAMP-2a. This may result in the reduction of the stability of LAMP-2a, ultimately interfering with the CMA.

In addition, the relation between the LAMP-1 and LAMP-2a was accidentally observed in our study. The LAMP-1 is a major protein component of the lysosomal membrane. The LAMP-1 has 37% amino acid sequence homology with LAMP-2, which is also the protein component of the lysosomal membrane [44]. LAMP-2a is not only an important active subtype of LAMP-2, but also the speed-limiting protein of the CMA metabolic pathway. LAMP-1 deficiency can induce overexpression of murine LAMP-2a [45], while our study showed that inhibition of HDAC6 hindered the increase of LAMP-2a; as a consequence, the number of LAMP-1 was compensatory increased to resist HI-induced oxidative stress toxicity (Figure 5(c)). Therefore, we speculated there is a regulatory balance between LAMP-2a and LAMP-1 that can further regulate the CMA pathway, which needs further study to be proven.

In this study, we provided the evidence that SCI and HI stress activated the function of CMA pathway to restrain ROS generation and alleviate cell damage, while loss of HDAC6 hindered the CMA activity increase, which could partially regulate the acetylation of HSP90. Consequently, the deacetylation of HSP90 by HDAC6 is perhaps a potential therapeutic target in acute SCI model and arouses our interests to study it in depth.

## 5. Conclusions

Hypoxia-ischemia- (HI-) induced oxidative stress plays a role in secondary pathocellular processes of acute SCI due to hypoxia-ischemia (HI) from many kinds of mechanical trauma. Our results showed that inhibition of HDAC6 accelerated reactive oxygen species (ROS) generation and cell apoptosis in response to the HI. Deficiency of HDAC6 hindered the CMA activity to resistance HI-induced oxidative stress. Furthermore, our results provided the experimental evidence for the potential role of HDAC6 in the regulation of CMA by affecting HSP90 acetylation. Therefore, HDAC6 plays an important role in the function of CMA pathway under the HI stress induced by SCI and arouses our interests to pursue it further in an in-depth study.

## Abbreviations

SCI: Spinal cord injury
ROS: Reactive oxygen species
CMA: Chaperone-mediated autophagy
HDAC6: Histone deacetylase-6
HSP90: Heat-shock protein of 90 kDa
HSC70: Heat-shock cognate protein of 70 kDa
LAMP-2a: Lysosome-associated membrane protein type 2a
LAMP-1: Lysosome-associated membrane protein type 1
HI: Hypoxia-ischemia
HIF-1-alpha: Hypoxia-inducible factor 1 alpha
RNase A: Ribonuclease A
MMP: Mitochondrial membrane potential.
